# Validation of the patient-doctor relationship questionnaire: a cross-sectional study among exit patients in Ghana

**DOI:** 10.1186/s12875-026-03363-w

**Published:** 2026-05-23

**Authors:** Obed Sarpong Adusei, Oziegbe Oghide, Kathryn Spangenberg, Aisa Abba Gana, Kalala Kankonde Serge

**Affiliations:** 1 SDA Hospital, Asamang, Ashanti Region Ghana; 2 Family Medicine Directorate, Komfo Anokye Teaching Hospital, Kumasi, Ghana; 3 Accident and Emergency Department, Lagos University Teaching Hospital, Lagos, Nigeria; 4 Family Medicine Department, Aminu Kano Teaching Hospital, Kano, Nigeria; 5 Family Medicine Department, Protestant University of Congo, Kinshasa, Kinshasa Democratic Republic of the Congo

**Keywords:** Doctor-Patient relationship, Patient-reported, PDRQ-9, Komfo Anokye Teaching Hospital, Ghana

## Abstract

**Background:**

The patient-doctor relationship is an essential component of patient-centered care. But there are limited tools available for assessment, especially in the low- and middle-income countries, including Ghana. This study aimed to validate the Patient-Doctor Relationship Questionnaire-9 (PDRQ-9) in Ghana.

**Methods:**

A cross-sectional validation study was conducted among 366 exit patients aged ≥ 18 in October 2023 at the Family Medicine Directorate Outpatient Clinic of the Komfo Anokye Teaching Hospital in Ghana. After taking the PDRQ-9 through adoption, face validation, and field testing, factor analysis and internal consistency testing were done.

**Results:**

At a significant Bartlett’s test of sphericity, *p* < 0.001, the Kaiser-Meyer-Olkin value was 0.94. Principal Component Analysis yielded only one component factor, which explained 81% of the variance. One eigenvalue exceeded 1.0. The bend of the scree plot was at the second factor, and one factor remained above the random values in parallel analysis. The factor loadings ranged between 0.4 and 0.97. The Cronbach’s alpha for the PDRQ-9 Ghana version was 0.96.

**Conclusion:**

The PDRQ-9 Ghana version is a valid and reliable unidimensional scale that can adequately measure patient satisfaction with their relationship with primary care physicians in the study population. It can serve as a tool for quality improvement.

**Supplementary Information:**

The online version contains supplementary material available at 10.1186/s12875-026-03363-w.

## Introduction

The doctor-patient relationship is instrumental in achieving two of the goals of Universal Health Coverage (UHC), namely, improved health outcomes and responsiveness, as better expressed by the patient [1, 2]. However, both doctors and patients have different opinions on the relationship [3]. While patients emphasize the interpersonal skills of physicians, the latter consider the pivotal roles of competence, trust, and technical expertise in the doctor-patient relationship [4]. Patients rate encounters poorly in contrast to the view of doctors [5]. Also, many patients do not trust their doctors [5].

Again, while patients of higher educational levels find doctors who are “sensitive to emotions” more appealing, those with low levels of education prefer doctors with good interpersonal relationships [6]. These situations highlight a mismatch of factors to be considered in relationship building between doctors and their patients, which affects continuity of care [4]. The doctor-patient relationship is affected by trust and confirmation of patients’ expectations [7]. Interestingly, the patients’ level of satisfaction is irrespective of the place where the care is given [8].

Despite the difference in the agenda of both patients and their doctors, the achievement of a common ground improves outcomes [3]. Patients generally expect a positive doctor-patient relationship and so focus more on factors leading to that objective [9]. Their evaluation of the doctor-patient relationship is influenced by patients’ overall trust [10], and they are more satisfied with doctors who educate their patients on disease prevention and the side effects of drugs [8]. Communication, therefore, is the bedrock of the doctor-patient relationship, and a language barrier has detrimental effects on the relationship [11].

The doctor-patient relationship is improved by a long-term relationship, as it improves the doctor’s knowledge of the patient’s medical history [12]. Other studies also emphasize improvement in patient satisfaction where there is shared decision-making, comprehensible communication, and provider empathy [13, 14].

A friendly patient-doctor relationship is an indispensable precondition in this age of patient-centered care [2]. Consequently, it is necessary to make a conscious effort to guarantee a positive doctor-patient interaction through assessments.

Physicians are increasingly using Patient-Reported Outcome Measures (PROMs), Patient Reported Experience Measures (PREMs), and Patient Satisfaction Surveys as avenues to improve screening, shared decision-making, patient management, and monitoring [15–19].

Self-reported patient satisfaction is a quality improvement measure of the health system. This can be achieved using the Patient-Doctor Relationship Questionnaire 9 (PDRQ-9). The PDRQ-9 tool is a short, validated instrument that assesses the level of trust, communication, support, and empathy during a therapeutic encounter to estimate the overall level of patients’ satisfaction with an encounter.

Although the PDRQ-9 has been used in Europe, Asia, Latin America, and parts of Africa including Nigeria [20] and Ethiopia [21], information about its use in Ghana is limited. However, the PDRQ-9, when validated and adapted, may serve as a contextually relevant tool for measuring patient experiences in primary care and outpatient settings. This is especially important when managing chronic diseases because continuity of care and interpersonal trust have a direct impact on patient satisfaction, follow-up, and adherence. Also, it will be interesting to find out the case in Ghana, as the majority of patients do not have their designated primary care physicians to establish a long-term doctor-patient relationship. Therefore, the study aimed to validate the PDRQ–9 in the Ghanaian outpatient setting.

## Methods

### Setting

The study was conducted at the Family Medicine Directorate, the primary health care wing of the Komfo Anokye Teaching Hospital (KATH). KATH is the second-largest teaching hospital in Ghana, and is affiliated with the Kwame Nkrumah University of Science and Technology School of Medicine and Dentistry. It is a 1,200-bed capacity facility located in the Middle Belt of Ghana. The Family Medicine Directorate provides general outpatient care, emergency services, and serves as a transit point for patients seeking specialist care without a prior referral from the initial facilities visited.

### Study design

**Fig. 1:**
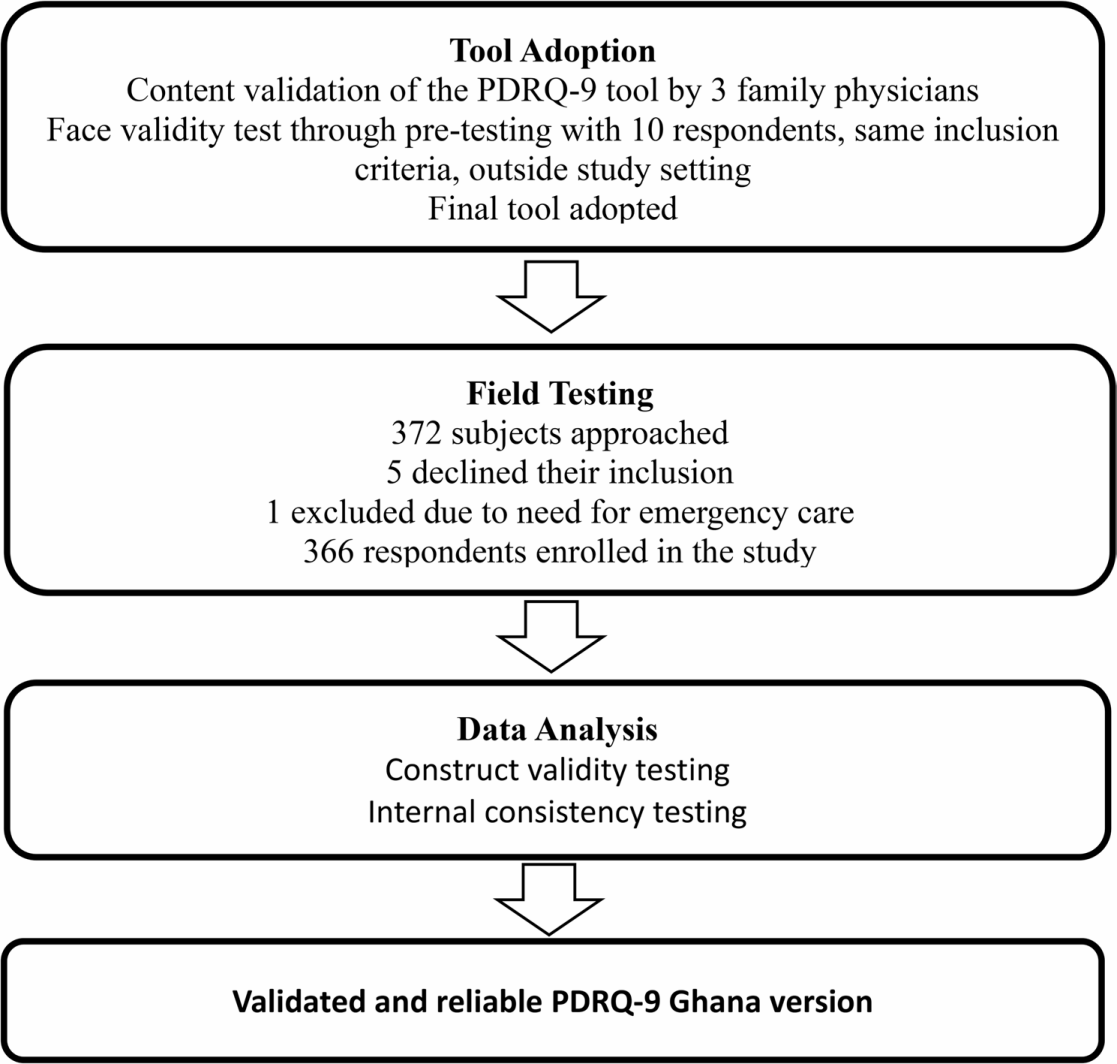
Flow chart of the conduct of study

This was a cross-sectional study conducted to validate the PDR Questionnaire among exit (just been seen) patients in the primary care outpatient department, shown in Fig. 1.

### The instrument for data collection

The study questionnaire (supplementary file) consisted of two parts. The first part covered the characteristics of respondents, including their age, gender, partnership status, educational level, and employment, whilst the second part covered an adapted PDRQ-9 [22], an already validated tool for doctor-patient relationship assessment in the primary care setting. The tool was validated in the Netherlands among adult primary care patients visiting general practice clinics in English with a Cronbach alpha of approximately 0.94. It uses a 5-point Likert scale for scoring the 9-point questionnaire, with 1 denoting “not at all appropriate” and 5 denoting “totally appropriate.” It provides a raw total score between 9 and 45 [23]. The greater the score, the more favorable the doctor-patient connection was perceived to be by the patient. The brevity and validity of the tool have improved its reception by patients across cultures, such as in Germany [24], Brazil [14], and China [25].

Content validity of the tool was done through a review by three family physicians, who formed an expert panel to assess the relevance of the items to the concept being measured and their application in the study setting. The panel had at least 5 years of experience each in primary care consultations. Content validation of the tool was done qualitatively. No quantitative indices were used. Also, the panel had no involvement with the study. No changes were made after content validation. Following that, a face validity test was done through a pretest of the questionnaire among 10 patients in a non-study hospital using the same inclusion criteria for the actual study to check the respondents’ understanding, interpretation, and scoring of the tool. Since the tool was in English, English language fluency was a strict criterion for face validity testing. No challenges were identified after testing. This resulted in a whole tool adoption without any change of wording of the original items or the scoring. The adopted tool was taken to the field for testing.

### Study population

Participants were patients who sought medical care at the study site in October 2023. All patients 18 years and above who exited from the consulting rooms were eligible for the study. Patients who could communicate independently and gave written consent were included in the study. Patients who required emergency care were exempted.

### Sample size calculation and sampling technique

Hogarty et al., [26] suggested a minimum of 3:1 to 20:1 items to respondents as a rule of thumb in tool validation. Using a ratio of 20:1, a minimum sample size of 180 was calculated for this study. However, to be able to validate the tool using factor analysis in our setting, a minimum sample of 200–300 was required [27]. We doubled the minimum sample size, considering non-response and the number for factor analysis. Participants were selected using a systematic sampling method. On a typical weekday (Monday to Friday), the directorate sees 200 patients at the outpatient clinics. We administered questionnaires to 10% of the total attendance. The sampling interval was calculated by dividing the average OPD attendance (200) by the average daily sample size of 10% (20), giving a sampling interval of ten (10). The first patient was chosen at random, and the 11th person after the first chosen was the second respondent, with an interval of 10, and so on till the total count for the day. If a patient was approached and refused to participate in the study or was deemed ineligible, the next patient was included.

### Study variables

Both the socio-demographic information (seven items) and the PDR Questionnaire (9 items) of eligible participants served as the independent variables for the study. The socio-demographic factors included the age, sex, educational level, partnership status, and employment status of participants, how long they had been attending the facility, and whether they had a primary care physician. The 9 items on the PDR Questionnaire can be found in Table 3. The dependent variable is the level of patient satisfaction with the patient-doctor relationship experienced.

### Data collection

Before the data was collected, the research assistants were offered a 1-day training. This was to ensure that they were familiar with the study protocol and understood their role. Data was collected over a month (October 2023) to minimize the possibility of a respondent giving a double response to the question. Participants responded to the questionnaire in the absence of their primary care physicians (PCP) in an office to ensure privacy and a true reflection of their experiences.

### Data management and analytical procedures

Data was initially entered into a spreadsheet and later exported into Stata version 16 before coding for analysis. Descriptive statistics, including frequencies, percentages, means, and standard deviations, were generated from the demographic characteristics to describe the variables of interest. The scores of the response were treated as a continuous variable, and the mean item score, as well as the mean total score, with their standard deviations, were calculated [28].

Three family physicians reviewed the content to confirm the item’s ability to cover the construct. Face validity was assessed with ten eligible respondents to evaluate clarity and understanding through a pilot test at a primary hospital close to the study site. No issues were detected. Field testing was done.

Sample adequacy was checked using the Kaiser-Meyer-Olkin (KMO) value > 0.6. We then determined the Bartlett’s test of Sphericity as an indication for exploratory factor analysis at *p* < 0.05 [29]. Factors were extracted. Initially, Principal Component Analysis was done to assess the underlying structure among the items. The number of factors was determined using eigenvalues, factor loadings above 0.4, and scree plot inspection. Rotation analysis was not done because only one component factor was exhibited. Again, we used parallel analysis to identify the optimal number of items to be retained empirically [30]. A 60% variance explanation was set to provide good empirical support for construct validity [31]. Due to the categorical nature of the tool indicators, inter-item and the preferred item-total correlation coefficients were determined [32]. Cronbach’s alpha of 0.7 and above [33] was used to assess the internal consistency of the tool items, the reliability, and the unidimensional construct validity of the PDRQ-Ghana version. Construct validity from factor structure was demonstrated.

### Ethical considerations

Ethical approval (KATH IRB/AP/150/23) was obtained from the Institutional Review Board of Komfo Anokye Teaching Hospital. Additionally, this study was done per the Helsinki Declaration. Permission was sought from the Family Medicine Directorate, where the data was collected. Before patients were included in the study, the purpose of the study was explained to them verbally. They received confidentiality guarantees, were told they may leave the study at any moment, and were free to refuse to answer any questions. Every responder was informed that filling out the survey indicated their informed agreement to the use of their information for research. All participants registered written voluntary approval on the anonymized questionnaire before finally responding to it.

## Results

### Participant characteristics

The participants’ ages ranged from 18 to 88 years, with a mean (standard deviation) age of 50.57 (± 16.03). The majority were over 50 years old (52.73%). About 68.58% were females, and 62.84% were married. The highest education level of more than a third of the participants (47.81%) was basic education. Similarly, more than a third (44.54%) were self-employed. Additionally, the majority (70.77%) had attended KATH for less than 5 years, and almost all (97.54%) did not have a self-primary care physician (Table 1).

**Table 1:** Characteristics of respondents. Kumasi, Ghana, 2023

Variable	Frequency (*n* = 366)	Percentage (%)
Age group (years)		
≤ 30	50	13.66
31 to 40	55	15.03
41 to 50	68	18.58
51 to 60	88	24.04
61 to 70	68	18.58
> 70	37	10.11
Gender		
Male	115	31.42
Female	251	68.58
Educational level		
None	27	7.38
Basic	175	47.81
Secondary	105	28.69
Tertiary	59	16.12
Marital status		
Single	68	18.58
Married	230	62.84
Divorced	12	3.28
Separated	4	1.09
Widowed	52	14.21
Occupation		
Civil servant	42	11.48
Self-employed	163	44.54
Unemployed	145	39.62
Others ^α^	16	4.37
Number of years of hospital attendance		
< 5	259	70.77
≥ 5	107	29.23
Having a self-primary care physician		
Yes	9	2.46
No	357	97.54

α = artisans, commerce, and students

### Sampling adequacy

The Kaiser-Meyer-Olkin value was 0.94, indicating that the sample was highly adequate for factor analysis. Additionally, Bartlett’s test of sphericity was significant with a *p*-value of < 0.001, showing that the data were suitable for factor analysis and that the correlation matrix significantly differed from an identity matrix. Both tests confirmed that the data are ideal for Exploratory Factor Analysis (Table 2).

**Table 2:** Test for sampling adequacy

Test	Statistic	*p*-value
Kaiser-Meyer-Olkin	0.94	-
Bartlett’s Test of Sphericity	X^2^(36) = 5034.01	< 0.001

### Factor structure of the PDRQ-9

The Exploratory Factor Analysis showed one dominant factor (Factor 1) with an eigenvalue of 7.29, far exceeding the Kaiser criterion (eigenvalue > 1). This factor alone explains 81.1% of the total variance, indicating that the items cluster tightly around one core dimension. Factors 2 to 9 had eigenvalues < 1, signifying that they do not meaningfully contribute to the explained variance (Table 3).

**Table 3:** Eigenvalues and total variance explained for PDRQ-9 Items (*n* = 366)

Variable	Eigenvalue	Difference	proportion	Cumulative
Factor 1	7.29	6.44	0.8109	0.8109
Factor 2	0.85	0.62	0.0949	0.9058
Factor 3	0.23	0.06	0.0255	0.9314
Factor 4	0.17	0.03	0.0191	0.9505
Factor 5	0.14	0.03	0.0160	0.0665
Factor 6	0.11	0.03	0.0124	0.9789
Factor 7	0.08	0.01	0.0091	0.9880
Factor 8	0.07	0.03	0.0076	0.9956
Factor 9	0.04	-	0.0044	1.00

LR test: independent vs. saturated: X^2^(36) = 5047.95, *p* < 0.001

**Fig. 2:**
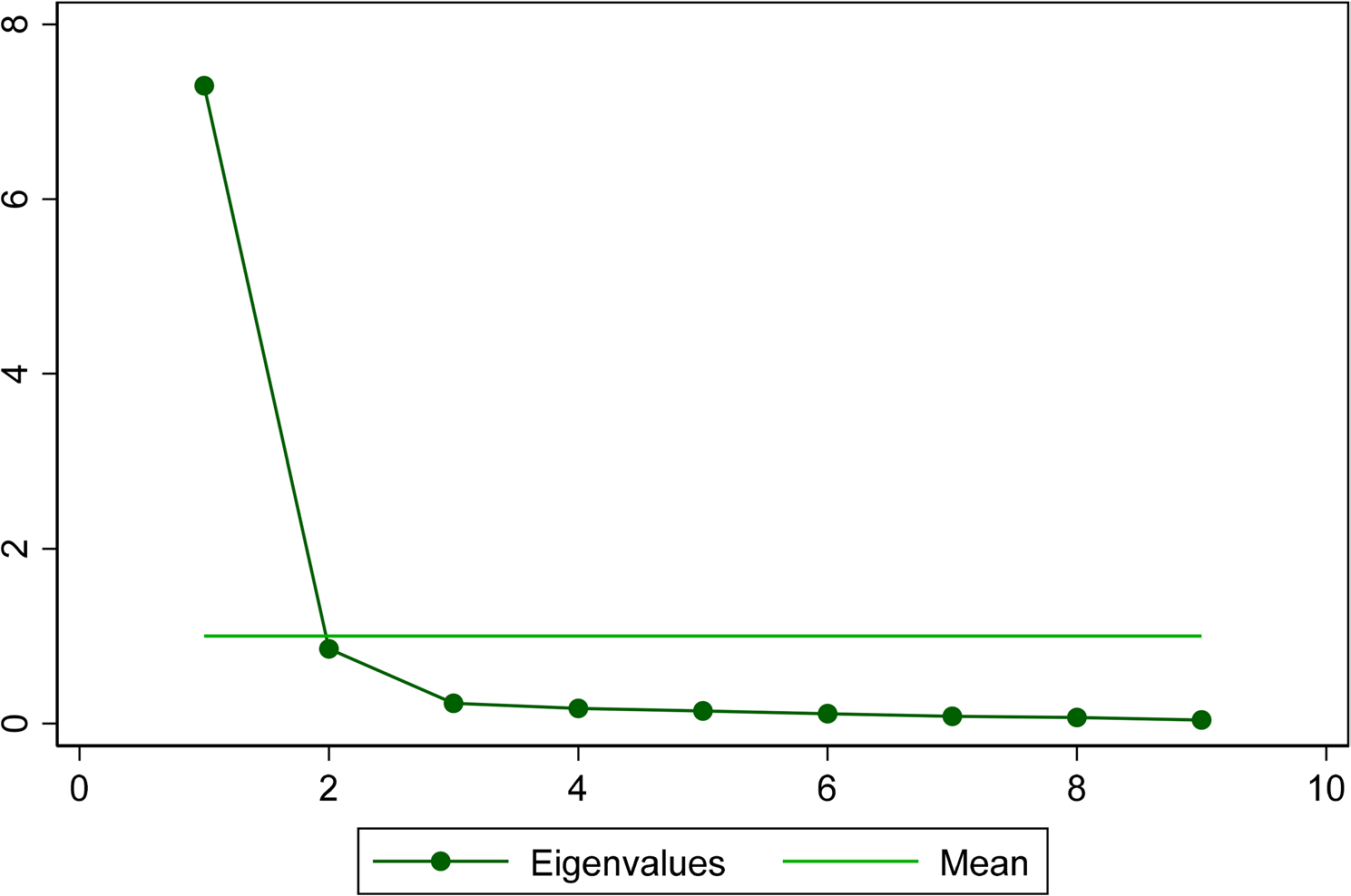
Scree plot of eigenvalues after factor

**Fig. 3:**
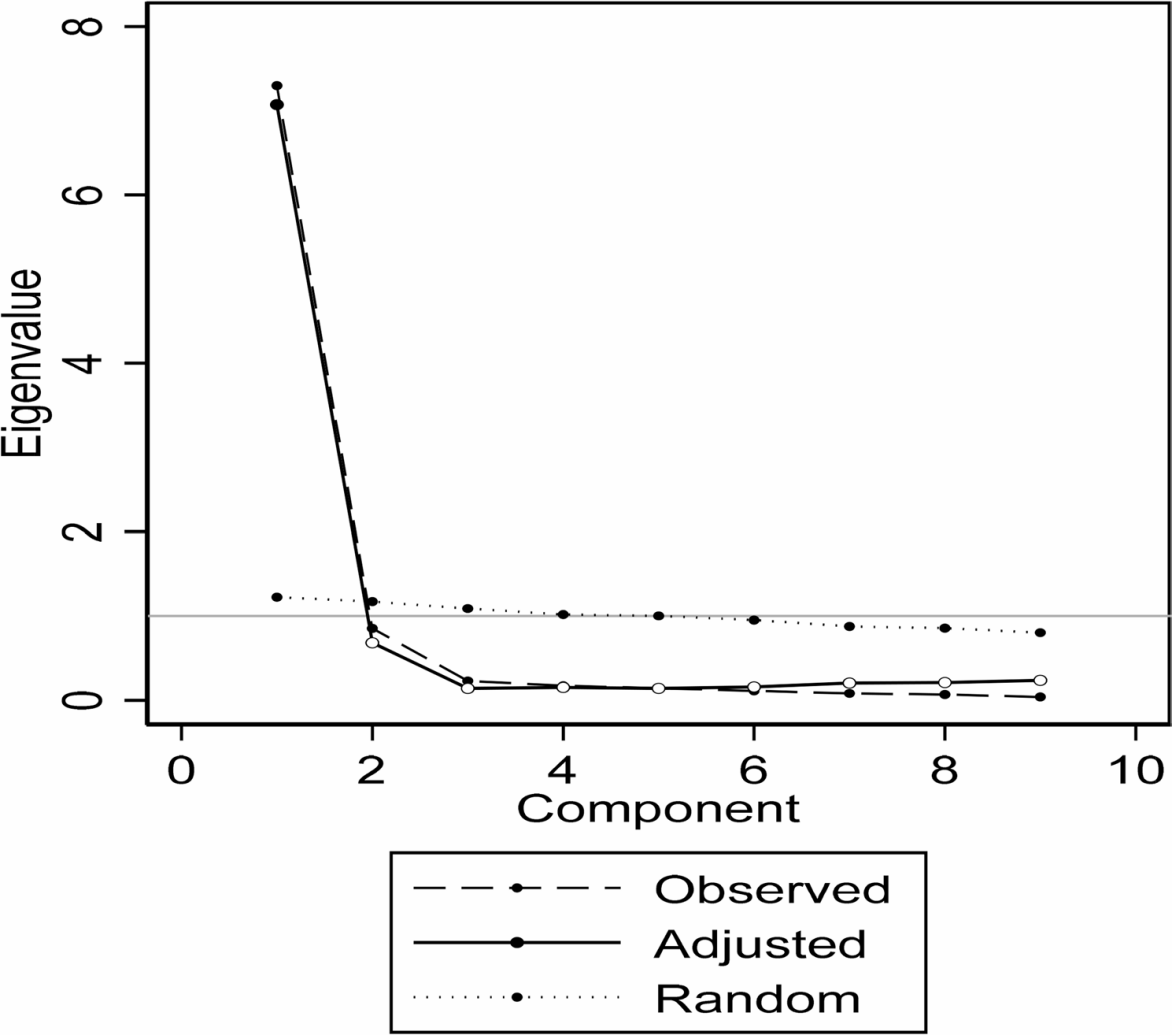
Parallel analysis of PDRQ-9 items

Additionally, the scree plot displayed a steep decline from Factor 1 to Factor 2. A pronounced “elbow” was observed after the first factor. This suggests that only one factor should be retained. This pattern demonstrates a unidimensional structure of the PDRQ-9 scale (Fig. 2). The parallel analysis confirmed that only the first factor exceeded its corresponding random eigenvalue. All other eigenvalues fell below the randomly generated eigenvalues. This outcome also showed that only a single factor should be retained, further supporting a one-factor solution (Fig. 3).

The factor loadings ranged from 0.42 to 0.97. Eight (8) items loaded extremely high (> 90.0), suggesting a strong convergence on one underlying factor. However, the 9th item, “I find my PCP easily accessible”, showed a weak loading of 0.42, although acceptable, suggesting that it does not measure the same construct as strongly as the other 8 items. In addition, the uniqueness values for items 1 to 8 ranged from 0.062 to 0.147, indicating that only 6% to 14.7% of the variability in each item was due to measurement error. However, item 9 showed a uniqueness value (0.827) above the threshold of < 0.40, suggesting that the factor hardly explains this item (Table 4). Furthermore, on average, over 81% of the variance in the items was explained by the factor. This suggests a very strong convergent validity (Table 5).

**Table 4:** Factor loadings and unique variances

Code	Item	Factor loadings	Uniqueness
PDRQ-9			
PDRQ-1	My PCP help me	0.926	0.142
PDRQ-2	My PCP has enough time for me	0.968	0.062
PDRQ-3	I trust my PCP	0.923	0.147
PDRQ-4	My PCP understand me	0.962	0.072
PDRQ-5	My PCP is dedicated to help me	0.950	0.098
PDRQ-6	My PCP and I agree on the nature of my medical symptoms	0.932	0.132
PDRQ-7	I can talk to my PCP	0.946	0.106
PDRQ-8	I feel content with my PCP treatment	0.941	0.115
PDRQ-9	I find my PCP easily accessible	0.416	0.827

*PCP *primary care physician; Composite reliability (*CR*) = 0.974; Average Variance Extracted (*AVE*) = 0.811

**Table 5:** Descriptive statistics and reliability analysis of PDRQ-9 item (*n* = 366)

Item	Mean	SD	Corrected item-total correlation	Cronbach’s alpha if item is deleted
1. My PCP help me	4.26	0.97	0.889	0.950
2. My PCP has enough time for me	4.32	0.94	0.944	0.947
3. I trust My PCP	4.26	0.97	0.887	0.950
4. My PCP understand me	4.28	0.95	0.937	0.947
5. My PCP is dedicated to help me	4.27	0.94	0.924	0.948
6. My PCP and I agree on the nature of my medical symptoms	4.25	0.98	0.903	0.949
7. I can talk to My PCP	4.25	0.99	0.923	0.948
8. I feel content with my PCP treatment	4.27	0.99	0.906	0.949
9. I find My PCP easily accessible	3.40	1.34	0.367	0.983
Satisfaction index	4.17	0.88		

*PCP *primary care physician, *SD *standard deviation, Cronbach’s alpha = 0.958

Again, the PDR Questionnaire items 1 to 8 correlate strongly with each other, with a correlation coefficient (r) ranging from 0.83 to 0.96. However, item 9 had a weak correlation with all other items (Table 6). The strong inter-item correlation provides strong evidence for a unidimensional factor.

**Table 6:** Factor correlation matrix of the PDRQ-9 item (*n* = 366)

Variable	PDRQ-1	PDRQ-2	PDRQ-3	PDRQ-4	PDRQ-5	PDRQ-6	PDRQ-7	PDRQ-8	PDRQ-9
PDRQ-1	1								
PDRQ-2	0.919^**^	1							
PDRQ-3	0.835^**^	0.904^**^	1						
PDRQ-4	0.880^**^	0.942^**^	0.889^**^	1					
PDRQ-5	0.841^**^	0.891^**^	0.853^**^	0.922^**^	1				
PDRQ-6	0.837^**^	0.867^**^	0.806^**^	0.893^**^	0.905^**^	1			
PDRQ-7	0.847^**^	0.903^**^	0.833^**^	0.893^**^	0.897^**^	0.879^**^	1		
PDRQ-8	0.864^**^	0.908^**^	0.881^**^	0.872^**^	0.873^**^	0.853^**^	0.887^**^	1	
PDRQ-9	0.319^**^	0.346^**^	0.325^**^	0.348^**^	0.366^**^	0.369^**^	0.390^**^	0.312^**^	1

Bolden figures=factor loading

**Correlation is significant at the 0.01 level (2-tailed)

### Internal reliability

The corrected item-total correlation coefficients for the 9 items ranged from 0.367 to 0.944. Specifically, item-total correlation coefficients for items 1 to 8 (0.887 to 0.944) showed a very good consistency with the PDR Questionnaire construct; however, item 9 (0.367) was poor. The reliability analysis indicated a very good internal consistency (Cronbach’s α = 0.958) and an excellent reliability (Composite Reliability (CR) = 0.974) (Table 5).

## Discussions

This study aimed to perform a cross-sectional validation of the PDRQ-9 questionnaire to assess the stability and reliability of the factor structure of the instrument in the Ghanaian primary care setting. To the best of our knowledge, it is the first study of its kind in Ghana. The work by Boateng and his colleagues [32] served as the guide and inspiration for this work. Constructs such as satisfaction vary across different cultures. We considered Exploratory Factor Analysis (EFA) as an appropriate methodology to assess items grouping into meaningful factors and their consistency with theory.

### Instrument adaptation

The expert panel of family physicians accepted all nine items of the original tool during the content validity assessment. Again, the ten patients who took part in the face validity testing had no challenge with the wording, interpretation, or the score measurement. The tool was therefore adopted for field testing without any amendment, suggesting that Ghanaian patients interpret the items of the tool similarly to patients in other contexts.

### Factorial analysis of the PDRQ-9 Ghana version

The KMO and Bartlett’s test for sphericity showed enough evidence (0.94; *p* < 0.001) for factor analysis. We determined the number of factors by going through several steps. Firstly, through PCA, only one component factor was extracted. This gave a suspicion that the tool was unidimensional and measured a single construct of satisfaction, as expected. We avoided rotational analysis because only one factor was extracted. Orthogonal rotation analysis requires at least two component factors to be present [31]. The eigenvalue of component 1 was 7.23. This was the only eigenvalue above 1.0. That of components 2 through 9 were less than 1.0 each, indicating an insignificant contribution to the concept being measured. Furthermore, the screeplot of factor loadings inspection (Fig. 2) showed a steep slope between the first and second component factors, forming an elbow at the second factor, and flattening afterwards. Again, we used parallel analysis [30] to identify the optimal number of items to be retained empirically, and it showed (Fig. 3) that only one factor was above the random values. Furthermore, from the factor loadings, all items loaded strongly on the single factor, demonstrating each item’s meaningful contribution to the overall construct, aligning with previous validations of the PDRQ-9 across the continent. The scree plot and parallel analysis together showed clearly a single-factor solution as per the original instrument and demonstrated the unidimensional measure of the patient–doctor relationship questionnaire. Though a 60% variance explanation was set [31] to provide good empirical support for construct validity, the single factor was able to explain about 81% of the variance, further stressing that the tool is unidimensional.

### Reliability and validity test of the PDRQ-9 Ghana version

The instrument exhibited excellent internal consistency, confirming the findings in the factor analysis. The corrected-item total correlation was more than 0.3 for all items, emphasizing the tool’s reliability [34] and all items’ contribution to the tool’s overall reliability. Furthermore, all inter-item correlations among items 1 through 9 were positive and significant (*p* < 0.001). Cronbach alpha was 0.96 above the 0.7 threshold [33], showing strong internal structure validity and internal consistency reliability of the tool. This was similar to the Brazil study [14], emphasizing its one-factor model and showing its acceptable internal consistency, validity, and reliability, endorsing its applicability in the Ghanaian setting. Similar findings were found in the Turkish (ɑ = 0.91) [35], German (ɑ = 0.95) [24], and American (ɑ = 0.96) [36] studies supporting cross-cultural stability and universality of the doctor-patient relationship perception and a justification for local use. All factor loadings of items were above 0.90 except item 9, which was 0.42. This may be due to a misunderstanding of the question [22] or the fact that patients do not have dedicated personal physicians assigned to them whom they can easily call to schedule an appointment in our setting. The robustness and psychometric reliability of the tool for assessing patients’ satisfaction of their relationships with doctors in Ghana was exhibited by the composite reliability (CR) values exceeding recommended thresholds. Based on the above, the construct validity of the PDRQ-9 Ghana version has been demonstrated.

### Clinical relevance

The instrument validation in the Ghanaian context has important implications for research and practice for the following reasons. Firstly, it serves as a reliable measure for doctor-patient relationship measurement, a central component in patient-centered care, having a bearing on overall treatment outcome and patient satisfaction. Secondly, it will serve as a quality improvement measure for clinicians, health managers, and policymakers for interpersonal quality assessment. Thirdly, the basis for future research is created for the measurement of the quality of the doctor-patient relationship in chronic disease management. Lastly, validating the tool in Ghana adds to the global evidence that the instrument has cultural adaptability and potential for cross-country comparisons.

### Limitations of the study

The PDRQ-9 used has contextual variability, making it susceptible to social desirability bias. Therefore, patients may have provided responses perceived to be socially acceptable. The face-to-face data collection may have influenced this as well. Also, the doctors’ perspective of the relationship was not assessed in this study; hence, the discrepancies between the patients’ and physicians’ perceptions cannot be analyzed. Again, this is a single facility study, and the findings cannot be generalized to other places. This is a cross-sectional validation study. Further studies with rigorous analysis, including confirmatory factor analysis, are recommended to establish the tool’s validity in diverse Ghanaian contexts.

## Conclusion

The PDRQ-9 Ghana version is an appropriate, valid, and reliable unidimensional scale that can adequately measure patient satisfaction with their relationship with primary care physicians in the study population. It can serve as a tool for quality improvement.

## Supplementary Information

Supplementary Material 1.

## Data Availability

Data are provided with the manuscript.

## Abbreviations

EFA: Exploratory Factor Analysis
KATH: Komfo Anokye Teaching Hospital
KMO: Kaiser–Meyer–Olkin
OPD: Outpatient Department
PCA: Principal Component Analysis
PCP: Primary Care Physician
PDRQ: 9–Patient–Doctor Relationship Questionnaire–9
PREMs: Patient–Reported Experience Measures
PROMs: Patient–Reported Outcome Measures
SD: Standard Deviation
