# Supplementary material for: Validation of the patient-doctor relationship questionnaire: a cross-sectional study among exit patients in Ghana
Source: BMC Prim Care. 2026 May 23;27:300. doi: 10.1186/s12875-026-03363-w (PMC13471460; doi:10.1186/s12875-026-03363-w)
Supplement: Supplementary file 1 [file 12875_2026_3363_MOESM1_ESM.pdf]

QUESTIONNAIRE FOR PATIENT-DOCTOR RELATIONSHIP PERCEPTION

Introduction and Consent:

We are studying the perception of patients on the Patient-Doctor Relationship in Ghana. The Komfo Anokye Teaching Hospital has been chosen as the study site. You are hereby, being requested to take part in responding to the following questions of your own free will.

You can decline your participation in this study at any point of the process and it will not affect any further management you will receive in this facility. To further show our commitment to ensure confidentiality your name is not needed in the collection of this data.

Do you agree to take part in the study after this explanation? Yes: ____ Sign: __________

Demographics

Tick the appropriate box apart from age (write age in years)

Q/N	Variable	1	2	3	4	5	6
1	**Age**						
2	**Gender**	Male	Female				
3	**Education**	None	Basic	Secondary	Tertiary		
4	**Partnership status**	Single	Cohabiting	Married	divorced	separated	widowed
5	**Employment status**	Unemployed	Artisan	Commerce	Civil	Self-employed	

6. How long have you been attending the clinic here? Months ____ (if < 1 year) Years ____

7. Do you have a self-Primary Care Physician? 1. Yes ☐ 2. No ☐

PDRQ-9

Instruction:

You will read nine statements that a person can make about his/her Primary Care Physician (PCP). Please choose the appropriateness of each statement for your PCP by marking one number per statement. The meaning of the numbers is as follows:

1= not at all appropriate 2= somewhat appropriate 3= appropriate

4= mostly appropriate 5= totally appropriate

		**SCORE**				
**Q/N**	**ITEM**	1	2	3	4	5
1	My PCP helps me					
2	My PCCP has enough time for me					
3	I trust my PCP					
4	My PCP understands me					
5	My PCP is dedicated to help me					
6	My PCP and I agree on the nature of my medical symptoms					
7	I can talk to my PCP					
8	I feel content with my PC's Treatment					
9	I find my PCP easily accessible					
	**Total score**					

Note: PCP = Primary Care Physician
